# Bacterial, Phytoplankton, and Viral Distributions and Their Biogeochemical Contexts in Meromictic Lake Cadagno Offer Insights into the Proterozoic Ocean Microbial Loop

**DOI:** 10.1128/mbio.00052-22

**Published:** 2022-06-21

**Authors:** Jaspreet S. Saini, Christel Hassler, Rachel Cable, Marion Fourquez, Francesco Danza, Samuele Roman, Mauro Tonolla, Nicola Storelli, Stéphan Jacquet, Evgeny M. Zdobnov, Melissa B. Duhaime

**Affiliations:** a Department F.-A. Forel for Environmental and Aquatic Sciences, Earth and Environmental Sciences, University of Geneva, Geneva, Switzerland; b Department of Genetic Medicine and Development, University of Geneva, Geneva, Switzerland; c Swiss Institute of Bioinformatics, Geneva, Switzerland; d Department of Ecology and Evolutionary Biology, University of Michigan, Ann Arbor, Michigan, USA; e Institute of Microbiology (IM), Department for Environmental Constructions and Design (DACD), University of Applied Sciences and Arts of Southern Switzerland (SUPSI), Manno, Switzerland; f Department of Botany and Plant Biology, University of Geneva, Geneva, Switzerland; g Aix Marseille University, Université de Toulon, CNRS, IRD, MIO UM 110, Marseille, France; h Université Savoie Mont-Blanc, INRAE, UMR CARRTEL, Thonon-les-Bains, France; Corporación CorpoGen

**Keywords:** Lake Cadagno, Proterozoic, ancient ocean, meromictic, microbial loop, viral ecology

## Abstract

Lake Cadagno, a permanently stratified high-alpine lake with a persistent microbial bloom in its chemocline, has long been considered a model for the low-oxygen, high-sulfide Proterozoic ocean. Although the lake has been studied for over 25 years, the absence of concerted study of the bacteria, phytoplankton, and viruses, together with primary and secondary production, has hindered a comprehensive understanding of its microbial food web. Here, the identities, abundances, and productivity of microbes were evaluated in the context of Lake Cadagno biogeochemistry. Photosynthetic pigments together with 16S rRNA gene phylogenies suggest the prominence of eukaryotic phytoplankton chloroplasts, primarily chlorophytes. Chloroplasts closely related to those of high-alpine-adapted Ankyra judayi persisted with oxygen in the mixolimnion, where photosynthetic efficiency was high, while chloroplasts of *Closteriopsis*-related chlorophytes peaked in the chemocline and monimolimnion. The anoxygenic phototrophic sulfur bacterium *Chromatium* dominated the chemocline along with *Lentimicrobium*, a genus of known fermenters. Secondary production peaked in the chemocline, which suggested that anoxygenic primary producers depended on heterotrophic nutrient remineralization. The virus-to-microbe ratio peaked with phytoplankton abundances in the mixolimnion and were at a minimum where *Chromatium* abundance was highest, trends that suggest that viruses may play a role in the modulation of primary production. Through the combined analysis of bacterial, eukaryotic, viral, and biogeochemical spatial dynamics, we provide a comprehensive synthesis of the Lake Cadagno microbial loop. This study offers a new ecological perspective on how biological and geochemical connections may have occurred in the chemocline of the Proterozoic ocean, where eukaryotic microbial life is thought to have evolved.

## INTRODUCTION

Roughly 2.3 billion years (Gyr) ago, the oceans were sulfide rich (1) and anoxygenic photosynthesis was a dominant mode of microbial primary production (2). Following the great oxidation event 2.4 to 2.1 Gyr ago (3), anoxygenic photosynthesis continued to sustain microbial life in the euxinic environments below oxygenated surface waters (1, 2, 4). Habitats in which anoxygenic photosynthesis significantly contributes to primary production continue to persist, such as in sulfur-rich meromictic lakes (5) like Lake Cadagno (6). These permanently stratified lakes are resistant to seasonal mixing events that would otherwise redistribute oxygen throughout the water column (7). In these systems, biogeochemical processes provide a link between the contrasting dominant modes of primary production, thereby directly connecting the food webs of the oxygenated and anoxic habitats. By resolving these biogeochemical connections, we may obtain insights into how oxygenic and anoxygenic photosynthesis may have evolved in the Proterozoic ocean’s chemocline.

Meromictic Lake Cadagno is a well-established ancient ocean model (8–12) owing to its permanently stratified oxygenated surface and euxinic bottom waters, its shallow chemocline within the photic zone, and sulfate and molybdenum concentrations lower than those of modern oceans. The microbial community composition of meromictic Lake Cadagno typifies the reconstructed signatures of life in the Proterozoic ocean (8, 10, 12). Purple and green sulfur bacteria (PSB and GSB, respectively) are among the hallmark bloom-forming microbes that are frequently observed in the chemocline of Lake Cadagno (13, 14). Within the chemocline, carbon, nitrogen, sulfur, and iron cycling are integrated through metabolic activities of the anoxygenic photosynthetic purple sulfur and green sulfur bacteria (15–18). Two PSB, Thiodictyon syntrophicum and Chromatium okenii, have been identified as the greatest contributors to primary production in Lake Cadagno (14, 16, 19). However, recent measurements of primary production have been limited to the chemocline (14, 20), and measurements of secondary production do not exist. Phytoplankton (including modern eukaryotes and cyanobacteria) likely coexisted with anoxygenic phototrophic sulfur bacteria in the Proterozoic ocean (21, 22). High phytoplankton abundances and productivity were expected in the oxic zone, because phytoplankton produces organic matter via oxygenic photosynthesis. In order to better understand microbial loop linkages across the stratified mixolimnion-chemocline transition in Lake Cadagno, paired measurements of productivity, microbial community composition, and diversity are essential next steps.

Lake Cadagno studies to date have focused on the phytoplankton and prokaryotic members of the microbial community and have yet to include viruses. Viruses are known to impact both phytoplankton and heterotrophic microbial populations through lysis and lysogeny and may hold a central position in the marine food webs and biogeochemical cycling (23, 24). Through the lysis of their microbial hosts, viruses play a role in the conversion of particulate organic carbon to dissolved organic carbon, which is predicted to be capable of sustaining the microbial loop (25, 26). Viruses have been identified in meromictic lakes (27–29), but their abundances across the vertical water column of Lake Cadagno are unknown.

To better understand the role the microbial loop plays in sustaining the food web across the stratified layers of Lake Cadagno, microbial community members (phytoplankton, prokaryotes, and viruses) were studied in the context of biogeochemical and primary and secondary production measurements. We hypothesized the mixolimnion to have low primary production relative to the chemocline, because the chemocline is known to be inhabited by a persistent microbial bloom of the primary producers phototrophic sulfur bacteria (14). We also expected high secondary production and viral abundance to be an indicator of effective organic matter recycling within the Lake Cadagno water column, because photoautotrophs, both phototrophic sulfur bacteria and phytoplankton, rely on heterotrophs and viruses for the remineralization of organic matter and nutrient cycling (30, 31). Through a combination of physical, chemical, and biological analyses, this work provides new evidence on how transitions between permanently stratified lake habitats and assemblages may sustain microbial food webs, informing our understanding of the aquatic ecosystems of early Earth.

## RESULTS

### Positions of the mixolimnion, chemocline, and monimolimnion.

**FIG 1:**
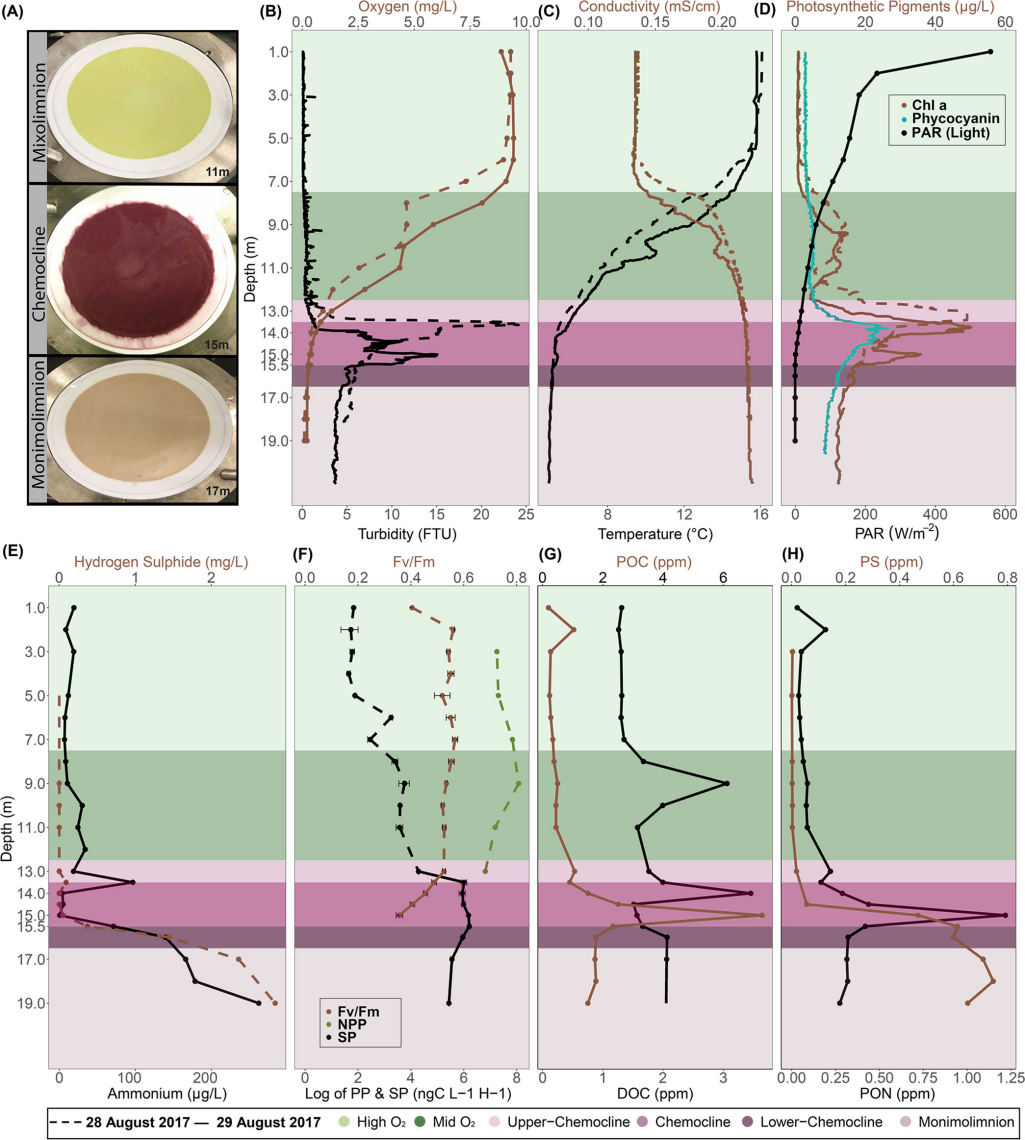
Depth profiles of biogeochemical parameters of Lake Cadagno. Dashed and solid lines represent 28 and 29 August 2017, respectively. Standard deviations for each sample are indicated by horizontal bars. (A) Photographs of biomass collected on 0.22-μm (142-mm-diameter) filters from the Lake Cadagno mixolimnion, chemocline, and monimolimnion strata. (B to F) Depth profiles of oxygen and turbidity (B), conductivity and temperature (C), Chl *a*, phycocyanin, and photosynthetically active radiation (PAR) (D), hydrogen sulfide and ammonium concentrations (E), net primary production (NPP) and secondary production (SP) rates (F), particulate organic carbon (POC) and dissolved organic carbon (DOC) concentrations (G), and particulate sulfur (PS) and particulate organic nitrogen (PON) concentrations (H). In panels B to H, the high O_2_ mixolimnion, mid O_2_ mixolimnion, low O_2_ mixolimnion-chemocline transition zone, chemocline, lower-chemocline, and anoxic monimolimnion layers are indicated by light green, dark green, light lilac, lilac, dark lilac, and light brown backgrounds, respectively.

Transitions between the major stratified layers sampled from Lake Cadagno were visually apparent by the color of pigmented biomass captured on 0.22-μm filters (Fig. 1A). From the surface to a depth of 12.5 m, the oxygen levels fell from a maximum of 9.24 mg/L to 2.79 mg/L (Fig. 1B, light green, day 2). As hypoxia is defined as 2.00 mg O_2_/L (32), 12.5 m marks the boundary of the oxic mixolimnion zone and the upper chemocline (Fig. 1, top light purple). The chemocline was at a depth of 13.0 to 15.5 m, as determined based on near-zero oxygen and light levels (day 2), peaked turbidity, a slight decrease in temperature, and a simultaneous rise in conductivity (Fig. 1B to D, dark purple). At 14.5 m, oxygen levels were near zero and <1% of surface light penetrated (Fig. 1B and D, day 2). Within the chemocline, the deep chlorophyll maximum (DCM) and peak turbidity shifted overnight between days 1 and 2, but in both cases, peak turbidity was observed 0.5 to 1.5 m below the DCM (Fig. 1B and D, day 2). Peak phycocyanin concentrations (26.53 μg/L) and basal fluorescence (*F_b_* = 66.0) were observed at the DCM (day 2, 13.5 to 14 m) and above a secondary chlorophyll peak at 15 m (Fig. 1D; see Fig. S2 in the supplemental material). The monimolimnion was defined as the zone between 15.5 m and the benthos. Gradual increases in particulate sulfur (0.74 ppm), H_2_S (2.84 mg/L), and ammonia (262.4 μg/L) concentrations were observed in the monimolimnion, as well as a decline in turbidity and photosynthetic pigments (Fig. 1B, E, and H).

10.1128/mbio.00052-22.3 FIG S2 Line plot depicting the results of the experiment to monitor the health of photosynthetic cells. A fast repetition rate fluorometer was used to measure basal fluorescence (*F_b_*; blue line) and maximum fluorescence (*F_m_*; black line), which were then used to calculate the maximum photosynthetic quantum yield (*F_v_*/*F_m_*; red line), a proxy for the health of photosynthetic cells. Download FIG S2, PDF file, 0.1 MB. Copyright © 2022 Saini et al. 2022 Saini et al. https://creativecommons.org/licenses/by/4.0/ This content is distributed under the terms of the Creative Commons Attribution 4.0 International license.

### Microbial production at the oxic-anoxic interface.

Between 7.0 and 11.0 m in the lower mixolimnion, there was a slight rise (up to 13.12 μg/L on day 1 and 11.80 μg/L on day 2) in chlorophyll *a* (Chl *a*) (Fig. 1D). The Chl *a* peak in the mixolimnion at 9.0 m corresponded with a peak in net primary production (NPP; 3,210 ng C L^−1^ h^−1^) (Fig. 1D and F). The NPP peak declined in the lower mixolimnion (11.0 m) and upper chemocline (13.0 m). Though NPP was not measured below 13.0 m, basal and maximum fluorescence measured through the chemocline gave an indication of phytoplankton photosynthetic activity and efficiency (Fig. S2). In the oxic-anoxic boundary, a maximum quantum yield (*F_v_*/*F_m_*, where *F_v_* is variable fluorescence and *F_m_* is maximal fluorescence) (an indicator of photosynthetic efficiency) of 0.49 was observed between 13.0 and 13.5 m and declined to 0.36 at 15.0 m (Fig. 1F), where the light was absent. Overlapping with this oxygenic photosynthesis, the highest rates of secondary productivity (SP) were observed through the chemocline (Fig. 1F). SP was significantly positively associated with some indicators of both photosynthetic microbes (Pearson correlation coefficient: Chl *a*, *R* = 0.96, *P* value = 1.7e−05; phycocyanin, *R* = 0.80, *P* value = 0.005) and total biomass (Pearson correlation coefficient: turbidity, *R* = 0.78, *P* value = 0.007; total cell counts, *R* = 0.65, *P* value = 0.04) (Fig. S3). The peak of dissolved organic carbon (DOC) (14.0 m) followed the peaks of particulate organic carbon (POC) and particulate organic nitrogen (PON) (15.0 m) (Fig. 1G and H). Below the chemocline, some organic and inorganic compounds continued to rise (particulate sulfur, H_2_S, NH_4_^+^), while PON and POC dropped (Fig. 1).

10.1128/mbio.00052-22.4 FIG S3 Grid and heat map depicting the Pearson correlation *R* coefficients and *P* values determined between data types; strong positive correlations are indicated by blue hues, strong negative correlations are indicated by red, and no correlation is indicated by yellow. Prefix of parameter name indicates the parameter type category. A, abundance: 16S rRNA amplicon-based relative abundances, flow cytometry-generated PLP and VLP counts, and phycobilisome-containing cell counts. D, diversity: alpha and beta diversity calculated from 16S-based genotypic and flow cytometry-based phenotypic Bray-Curtis distance measures. FCS, flow cytometry-derived data. N, nutrients, including DOC, POC, PON, PS, ammonium, and sulfate. P, physicochemical parameters from CTD, including light, conductivity, temperature, oxygen, Chl *a*, phycocyanin, turbidity. Secondary production is included with no prefix. Download FIG S3, PDF file, 0.6 MB. Copyright © 2022 Saini et al. 2022 Saini et al. https://creativecommons.org/licenses/by/4.0/ This content is distributed under the terms of the Creative Commons Attribution 4.0 International license.

### PLP and VLP abundances and trends.

**FIG 2:**
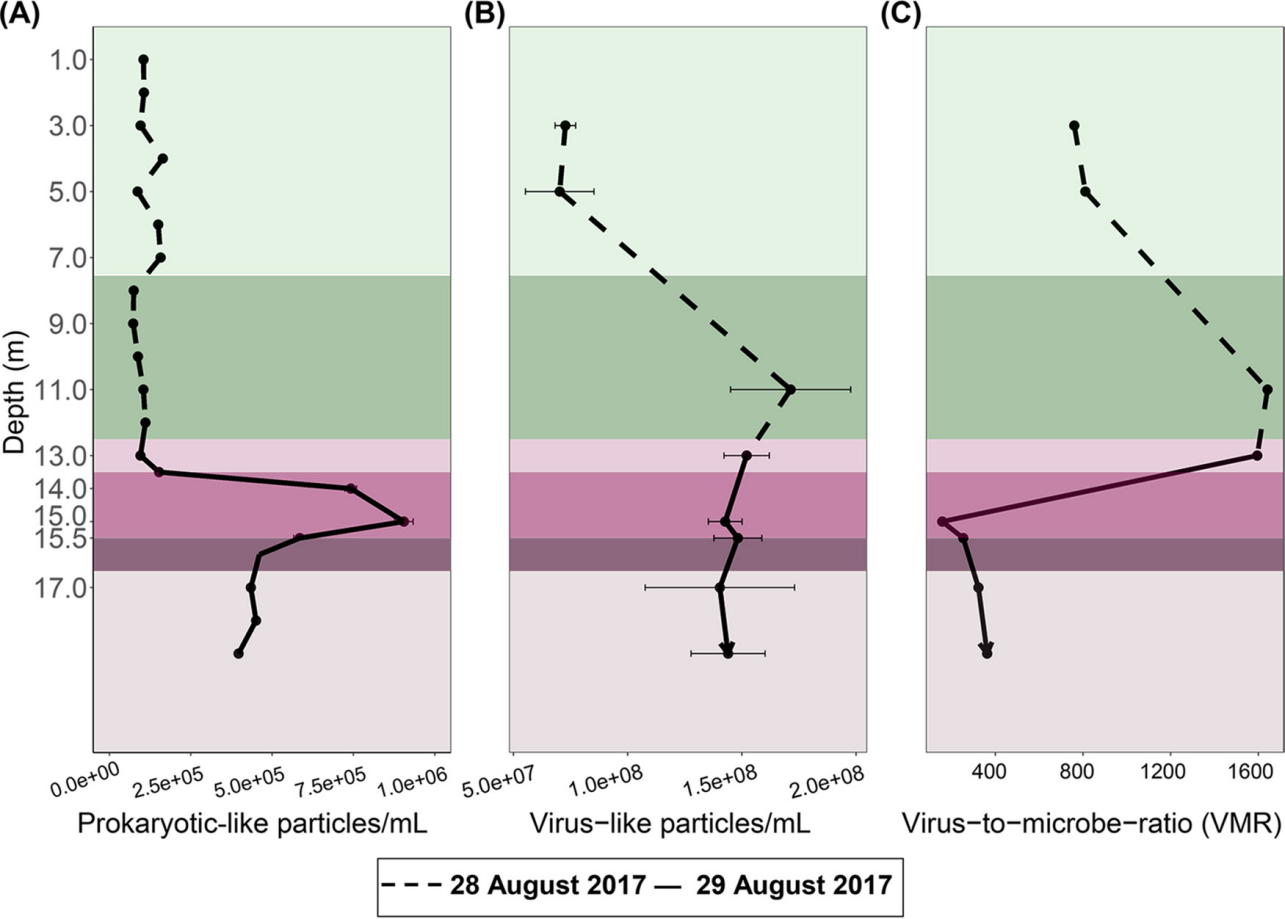
Dashed and solid lines represent 28 and 29 August 2017, respectively. Standard deviations for each sample are indicated by horizontal bars. (A to C) Abundances of prokaryote-like particles (A) and free virus-like particles (B) from flow cytometry analyses and the virus-to-microbe ratio as VLP/PLP (C).

The concentration of prokaryote-like particles (PLPs; prokaryotic cell counts inferred from flow cytometry [FCM] data) was highest in the chemocline at 15.0 m, roughly half that in the monimolimnion, and lowest in the mixolimnion (Fig. 2A). The flow cytometry PLP counts were in line with the fluorescent *in situ* hybridization (FISH) counts of the previous year (July 2016), which reported up to 10^6^ cells/mL in chemocline (33), and also concurred with the turbidity (Fig. 1D), which is used as a proxy for cell density in Lake Cadagno (12, 34). In contrast, free virus-like particles (VLPs; viral counts inferred from flow cytometry data) were highest at the bottom of the mixolimnion, lowest in the upper mixolimnion, and relatively invariable through the rest of the water column (Fig. 2B). As a result, the virus-to-microbe ratio (VMR) peaked at the bottom of the mixolimnion (1,642 at 11 m), but decreased drastically (158 at 15 m) when prokaryote-like particles rose in the chemocline (Fig. 2C).

### Bacterial community composition through the mixolimnion, chemocline, and monimolimnion.

**FIG 3:**
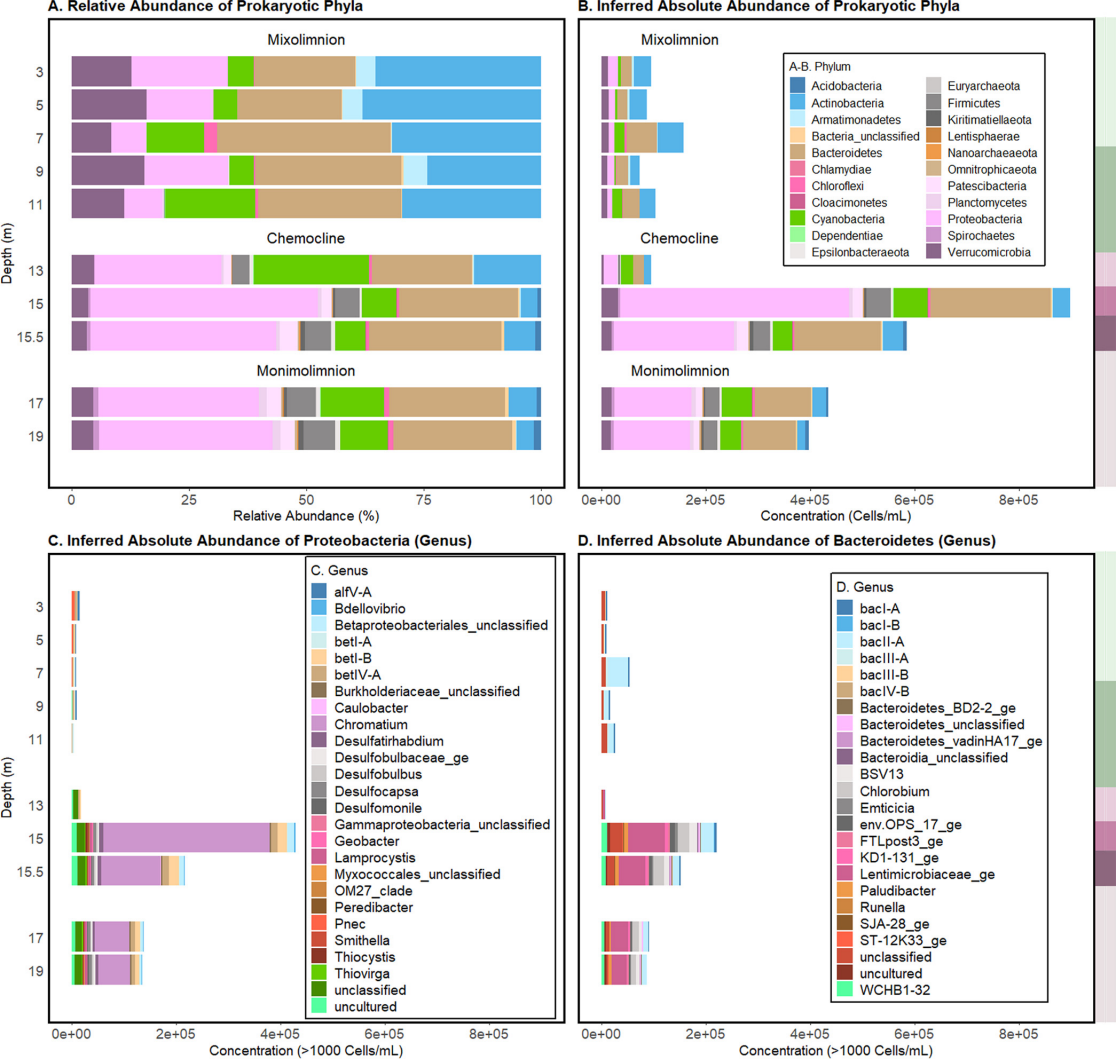
(A and B) Relative (OTU count scaled by total OTUs) (A) and inferred absolute (OTU count scaled by total FCM PLP counts [35]) (B) abundances of prokaryotic phyla at the sampled depths in Lake Cadagno. (C) Genera in the *Proteobacteria* phylum. (D) Genera in the *Bacteroidetes* phylum. To the right of each panel, the high O_2_ mixolimnion, mid O_2_ mixolimnion, low O_2_ mixolimnion-chemocline transition zone, chemocline, lower-chemocline, and anoxic monimolimnion layers are indicated by light green, dark green, light lilac, lilac, dark lilac, and light brown backgrounds, respectively, and correspond with the depths reported on the left-most *y* axis.

Both relative (operational taxonomic unit [OTU] count scaled by total OTUs) and inferred absolute (OTU count scaled by total FCM PLP counts [35]) abundances were considered to assess the abundances of microbial taxa through Lake Cadagno (Fig. 3A and B). Inferred absolute abundances of prokaryote-classified cells (“Bacteria” and “Archaea” OTU count scaled by total FCM PLP counts [35]) were at a minimum in the oxic mixolimnion (day 1, 0 to 12.5 m) (Fig. 3B; Table S1). On average, the mixolimnion community consisted primarily of the phyla *Actinobacteria* (35.37% of total OTUs; 33,841 cells/mL), *Bacteroidetes* (21.49% of total OTUs; 20,557 cells/mL), *Proteobacteria* (20.46% of total OTUs; 19,579 cells/mL), and *Cyanobacteria* (5.46% of total OTUs; 5,229 cells/mL) (Fig. 3A and B; Table S1).

10.1128/mbio.00052-22.1 TABLE S1 Relative abundances (OTU read count/total sample read count) and inferred abundances (relative abundances × FCM cell counts) of prokaryotic OTUs and chloroplasts. Download Table S1, XLSX file, 0.2 MB. Copyright © 2022 Saini et al. 2022 Saini et al. https://creativecommons.org/licenses/by/4.0/ This content is distributed under the terms of the Creative Commons Attribution 4.0 International license.

In the chemocline, the inferred absolute abundance of prokaryote-classified cells peaked at 15 m (Fig. 3A, day 2), which corresponded with peak turbidity, POC, and PON (Fig. 1). The major phyla represented at this depth were *Proteobacteria* (48.52%; 439,195 cells/mL) and *Bacteroidetes* (25.40%; 229,927 cells/mL). The genus that dominated these phyla at 15 m was the purple sulfur bacterium (PSB) *Chromatium* (35.25%; 319,051 cells/mL). *Chromatium* (*Proteobacteria*) was significantly positively correlated with turbidity (*R* = 0.93; *P* value = 7.25e−05) and total cell counts (*R* = 0.99; *P* value = 8.66e−09), suggesting its significant contribution to the chemocline biomass (Fig. S3). The inferred absolute abundances of an unclassified *Lentimicrobiaceae* OTU, the second most abundant OTU in the chemocline at 15 m, was significantly positively correlated with secondary production (*R = *0.8, *P* value *=* 5.09e−03), turbidity (*R* = 0.91, *P* value = 2.28e−04), and PLPs (*R* = 0.85, *P* value = 1.82e−03) yet negatively correlated with light (*R = −*0.78, *P* value *=* 7.33e−03) and oxygen concentration (*R = −*0.87; *P* value *=* 1.10e−03) (Fig. S3). Considering that *Lentimicrobium* species are known anaerobic fermenters (36), their strong correlation with secondary production and microbial biomass suggests their unknown contribution toward recycling of the chemocline biomass. Furthermore, though *Firmicutes* was not a dominant phylum itself, an unclassified genus of the family *Erysipelotrichaceae* (Firmicutes) (3.91%; 35,435 cells/mL), was among the abundant genera at 15 m (Fig. S4; Table S1). A green sulfur bacterium (GSB), *Chlorobium* (2.52% 20,849 cells/mL), was the fifth most abundant genus of the chemocline. The phototrophic sulfur bacteria (PSB) *Thiocystis*, *Thiodictyon*, and *Lamprocystis* and sulfate-reducing *Desulfocapsa* and *Desulfobulbus*, previously observed in Lake Cadagno (14, 17, 37), were present at less than 0.01% of the total OTUs in the chemocline (Table S1).

10.1128/mbio.00052-22.5 FIG S4 Inferred absolute abundances of bacterial genera through the water column of meromictic Lake Cadagno. Absolute abundances are inferred by multiplying the relative abundances (OTU read count/total sample read count) by FCM cell counts. Download FIG S4, PDF file, 0.01 MB. Copyright © 2022 Saini et al. 2022 Saini et al. https://creativecommons.org/licenses/by/4.0/ This content is distributed under the terms of the Creative Commons Attribution 4.0 International license.

On average, the total PLP counts in the monimolimnion (17.0 to 19.0 m) were less than half that of the peak chemocline counts and more than twice the average mixolimnion counts (Fig. 2A). The average relative and inferred (Fig. 3A and B) abundances of the dominant populations reflected those of the chemocline at 15 m, which included *Proteobacteria* (34.05% of total OTUs; 148,019 cells/mL), *Bacteroidetes* (24.65%; 107,165 cells/mL), *Cyanobacteria* (13.51% of total OTUs; 58,726 cells/mL), and *Actinobacteria* (5.84%; 25,425 cells/mL). In addition to bacteria, archaeal OTUs of the *Methanoregula* and *Woesearchaeia* genera were identified in the monimolimnion but in low abundances (<1,000 cells/mL) (Table S1), which may be partially attributed to primer bias against archaea in the 16S rRNA gene V4 amplification performed (38, 39).

### Phytoplankton in the mixolimnion, chemocline, and monimolimnion.

**FIG 4:**
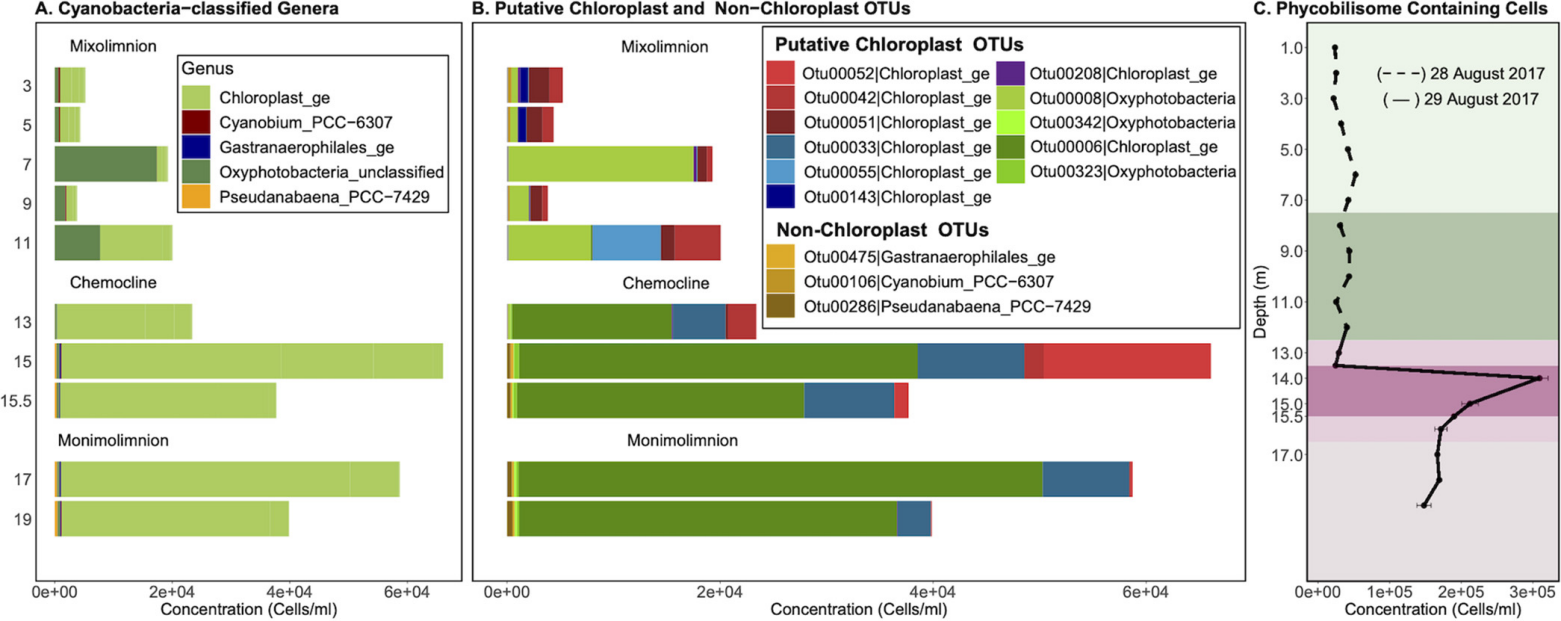
Abundances and phylogenies of Lake Cadagno phytoplankton based on 16S rRNA gene amplicon sequencing and flow cytometry. Mixolimnion samples (0 to 11.0 m) were collected on day 1, and chemocline and monimolimnion samples (12.0 to 19.0 m) were collected on day 2. Abundances in panels B and C are acquired by scaling 16S rRNA gene read counts by FCM cell counts. (A and B) Inferred concentrations (cells/mL) of OTUs were assigned to the phylum *Cyanobacteria*, and of those, the inferred concentrations (cells/mL) of OTUs were classified as putative chloroplasts and non-chloroplasts at the genus level (B). (C) Depth profile of phycobilin-containing cell counts based on flow cytometry with a 640-nm laser. The high O_2_ mixolimnion, mid O_2_ mixolimnion, low O_2_ mixolimnion-chemocline transition zone, chemocline, lower-chemocline, and anoxic monimolimnion layers are indicated by light green, dark green, light lilac, lilac, dark lilac, and light brown backgrounds, respectively.

*Cyanobacteria* were consistently among the abundant phyla at each depth, but at no depth did they dominate (Fig. 3A). Within this phylum, *Oxyphotobacteria* and chloroplast-classified OTUs dominated, representing 94 to 100% of the total *Cyanobacteria*-classified OTUs (Fig. 4A). Of the low-abundance cyanobacterial genera, *Cyanobium* was found throughout the water column, while *Pseudanabaena* and *Gastranaerophilales* were predominantly found at 15 m and deeper (Fig. 4A and B). To better understand the relationship between the microbial guilds of Lake Cadagno, we sought phylogenetic evidence to support the origin of these putative chloroplast OTUs, as described in previous literature (40, 41). The phylogeny of all *Cyanobacteria*-classified OTUs and their two nearest neighbors in SILVA (42) indicated that the chloroplast and *Oxyphotobacteria* OTUs clustered in clades with chloroplast 16S rRNA genes of cultured eukaryotic algae (Fig. 4B): Chlorophyta (Otu00008, Otu000342, Otu00006, Otu00323), Ochrophyta (Otu00033), Streptophyta (Otu00208), Haptophyta (Otu00143), and uncultured chloroplasts (Otu00042, Otu00051, Otu00052, Otu00055).

In the lower mixolimnion, between 7.0 and 11.0 m, where high oxygen, Chl *a*, and NPP persisted and peak *F_v_*/*F_m_* was observed (Fig. 1D and F), Otu00008, which is most closely related to the chloroplast of the cultured chlorophyte *Ankyra judayi*, dominated the subset of *Cyanobacteria*-classified OTUs, where they represented up to 89.97% of the total cyanobacterial OTUs of the mixolimnion (Fig. 4B and D). At 11.0 m, the next most abundant OTUs were Otu00042 and Otu00055, which belonged to a clade of chloroplasts from uncultured organisms, representing 21.53% and 31.63% of the total cyanobacterial OTUs, respectively.

**FIG 5:**
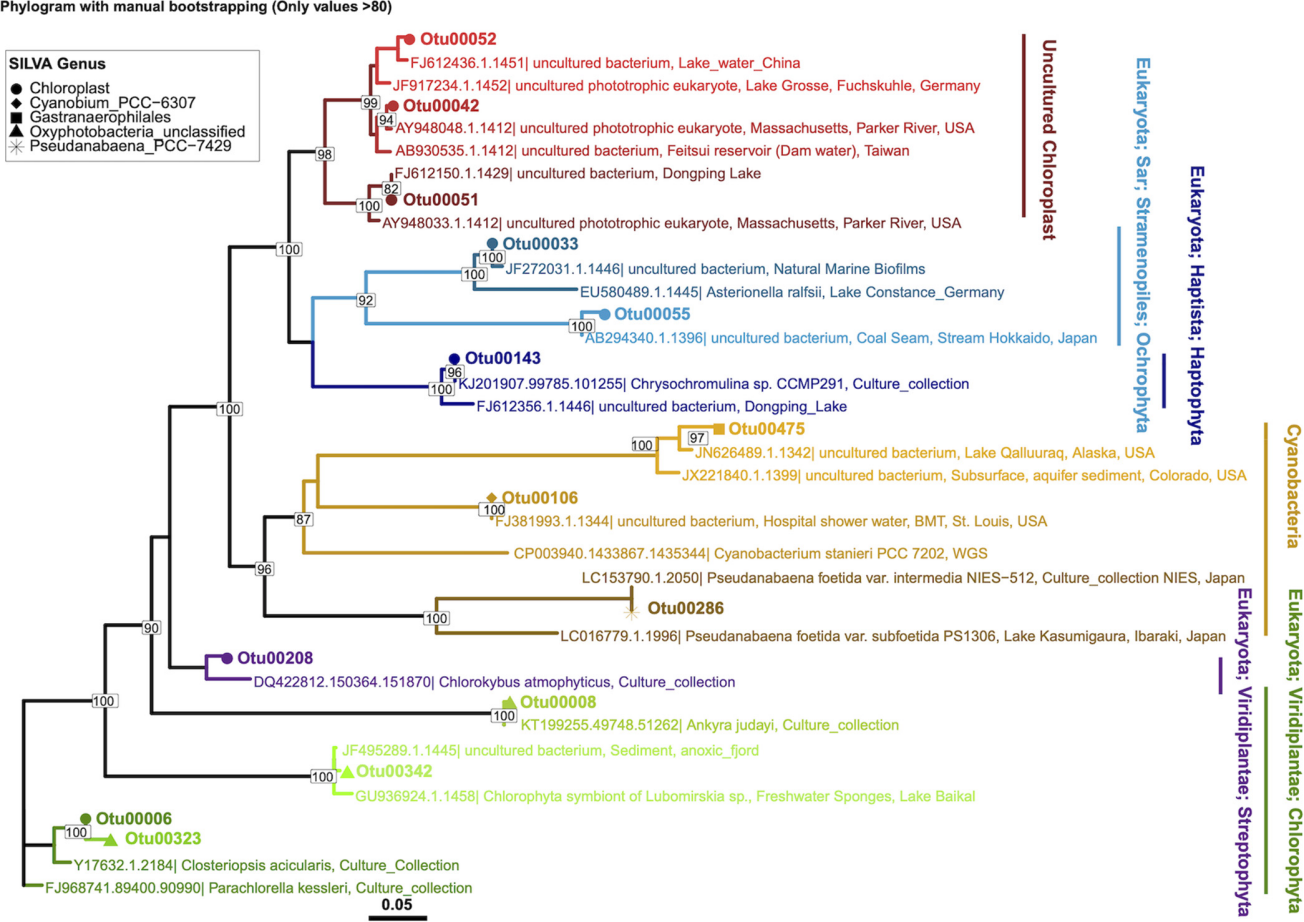
Phylogenetic tree of the representative 16S rRNA gene amplicon sequences of putative chloroplast and nonchloroplast OTUs shown in Fig. 4B assigned as *Cyanobacteria*, along with their two nearest neighbor sequences from the SILVA database. Clades are color coded consistently with the coloring of OTUs shown in the bar plot of Fig. 4B.

A primary chlorophyll peak was identified in the chemocline between 13.0 and 14.0 m, where FCM-based counts of phycobilin-containing cells (309,333 cells/mL) (Fig. 4C), phycocyanin pigments (26 μg/L, day 2) (Fig. 1D), and Chl *a* pigments (45 μg/L, day 2) rose sharply (Fig. 1D, day 2). While *Cyanobacteria*-classified OTUs represented nearly a quarter of all OTUs at the primary chemocline chlorophyll peak, they represented only 7.30% of the total OTUs at the secondary chemocline chlorophyll peak (15 m) (Table S1). The most abundant chloroplast OTU throughout the chemocline, Otu00006, was most closely related to the chloroplasts of the cultured chlorophytes *Parachlorella kessleri* and *Closteriopsis acicularis*, the latter genus (43) observed in meromictic Lake Tanganyika (Fig. 5). The next most dominant chloroplast-like OTUs, Otu00033 and Otu00052, clustered with cultured Ochrophyta chloroplasts and uncultured chloroplasts, respectively (Fig. 5). In the monimolimnion, Otu00006 and Otu00033 dominated the *Cyanobacteria*-classified OTUs (Fig. 5), which were most closely related to Chlorophyta and Ochrophyta chloroplasts, respectively.

### Prokaryotic genotypic and phenotypic diversity trends.

**FIG 6:**
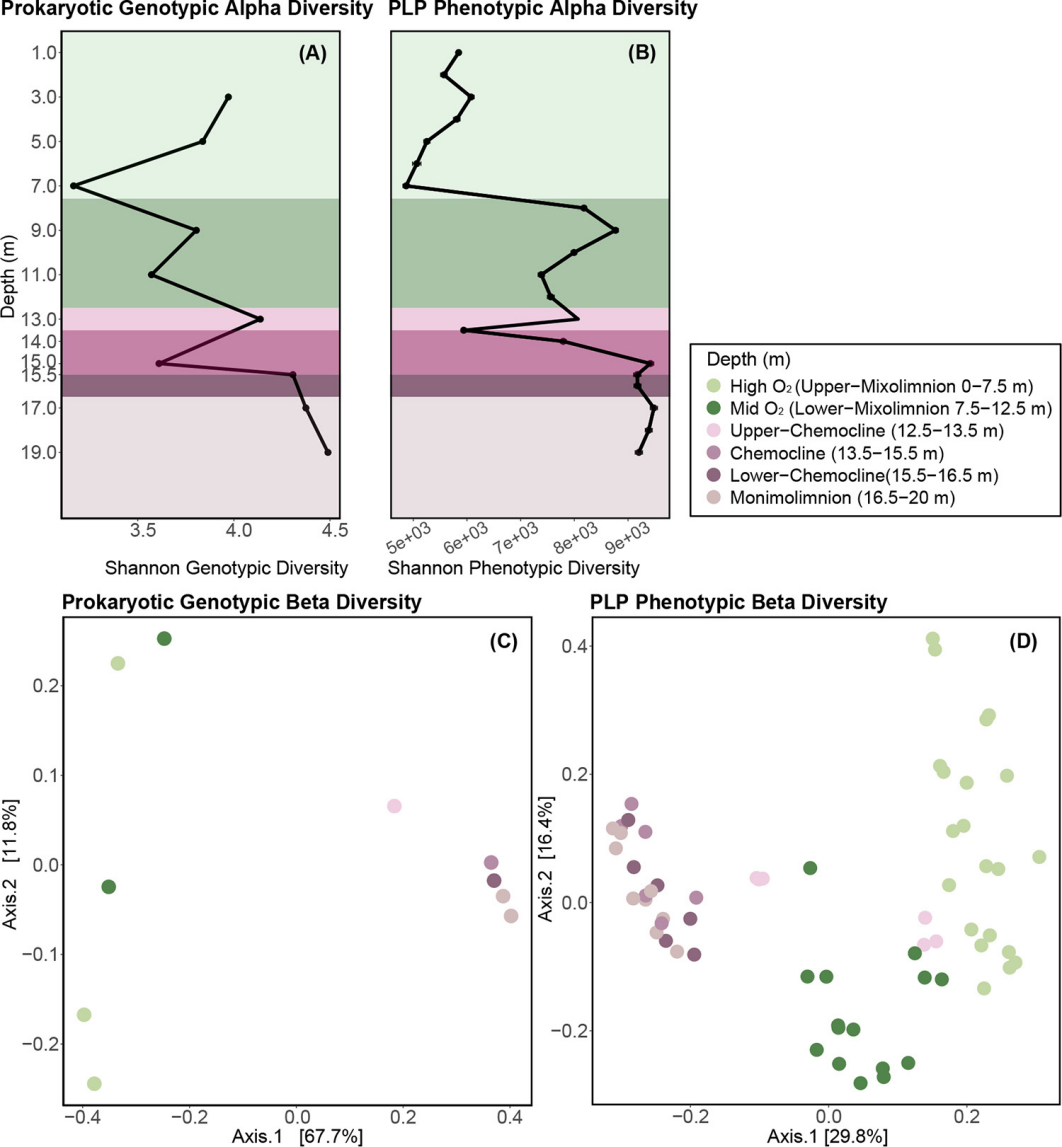
Trends in microbial community genotypic (16S rRNA gene amplicon sequencing, including prokaryotic and chloroplast-related OTUs) and phenotypic (PLP features based on flow cytometry [44]) alpha and beta diversity in Lake Cadagno. (A) Variation in genotypic alpha diversity (Shannon) with depth. (B) Variation in PLP phenotypic alpha diversity (Shannon). Mixolimnion samples (0 to 11.0 m) were collected on day 1, and chemocline and monimolimnion samples (12.0 to 19.0 m) were collected on day 2. Colors of data points represent the depth strata sampled. (C) PCoA represents the genotypic beta diversity dissimilarity (Bray-Curtis distance) of microbial communities. (D) PCoA represents the phenotypic beta diversity dissimilarity (Bray-Curtis distance) of microbial communities.

Bacterial genotypic diversity (16S rRNA gene) and PLP phenotypic diversity (based on individual cell features distinguished by FCM [44]) both dropped at 7.0 m and rose in the lower mixolimnion, where oxygenic primary production peaked (Fig. 6A and B; Fig. 1F). Overall, genotypic alpha diversity (Shannon) trends were nearly uniform, except at the peak of turbidity (15.0 m), where PLP phenotypic alpha diversity peaked and moderate genotypic alpha diversity was observed (Fig. 6A and B). Below the chemocline, consistently high genotypic alpha diversity and phenotypic alpha diversity were observed in the anoxic monimolimnion (Fig. 6). The increases in genotypic and phenotypic alpha diversity in the lower chemocline and monimolimnion were consistent with previous 16S rRNA gene-based studies of Lake Cadagno (33) and other permanently stratified systems, including Powell Lake (45), Ursu Lake (46), and Fara Fund Lake (46). Given the evidence that in microbial ecology “diversity begets diversity” (47), the observed increase in substrate diversity in the lower chemocline and monimolimnion (Fig. 1) likely facilitated greater microbial functional diversity, such as the anaerobic methane oxidation and phosphorus recycling described at those depths (8, 48). In addition, the increased particulate matter (Fig. 1) may have created greater habitat heterogeneity, which is also likely to lead to greater community diversity. Finally, it is also possible that the high diversity in the monimolimnion may have been partially attributed to the accumulation of sinking cells from the mixolimnion and chemocline, which may or may not still be living but contribute to community diversity measures.

In the principal-coordinate analysis (PCoA) ordinations of bacterial community dissimilarity (Bray-Curtis), the first two components combined accounted for 76.9% and 46.2% of all observed variation in genotypic and phenotypic beta diversity, respectively (Fig. 6C and D). When both genotypic diversity and phenotypic diversity were considered, the oxic mixolimnion and the coclustering anoxic chemocline and monimolimnion samples separated along the first axis (Fig. 6C and D). In the phenotypic diversity ordination (Fig. 6D), the mixolimnion samples separated along the second axis by whether they originated from the high-oxygen (1.0 to 7.0 m, light green) or the mid-oxygen (8.0 to 11.0 m, dark green) zone (Fig. 6C and D). As with phenotypic alpha diversity, oxygen and light were negatively correlated with phenotypic and genotypic beta diversity (Fig. S3).

## DISCUSSION

In this study, the investigation of viral, microbial, and biogeochemical spatial dynamics through Lake Cadagno’s water column improves our understanding of how biological and geochemical connections between the spatially segregated food webs in the Proterozoic ocean model may have manifested (2, 8, 10, 12).

### Eukaryotic algae associated with oxygenic phototrophy in the mixolimnion.

**FIG 7:**
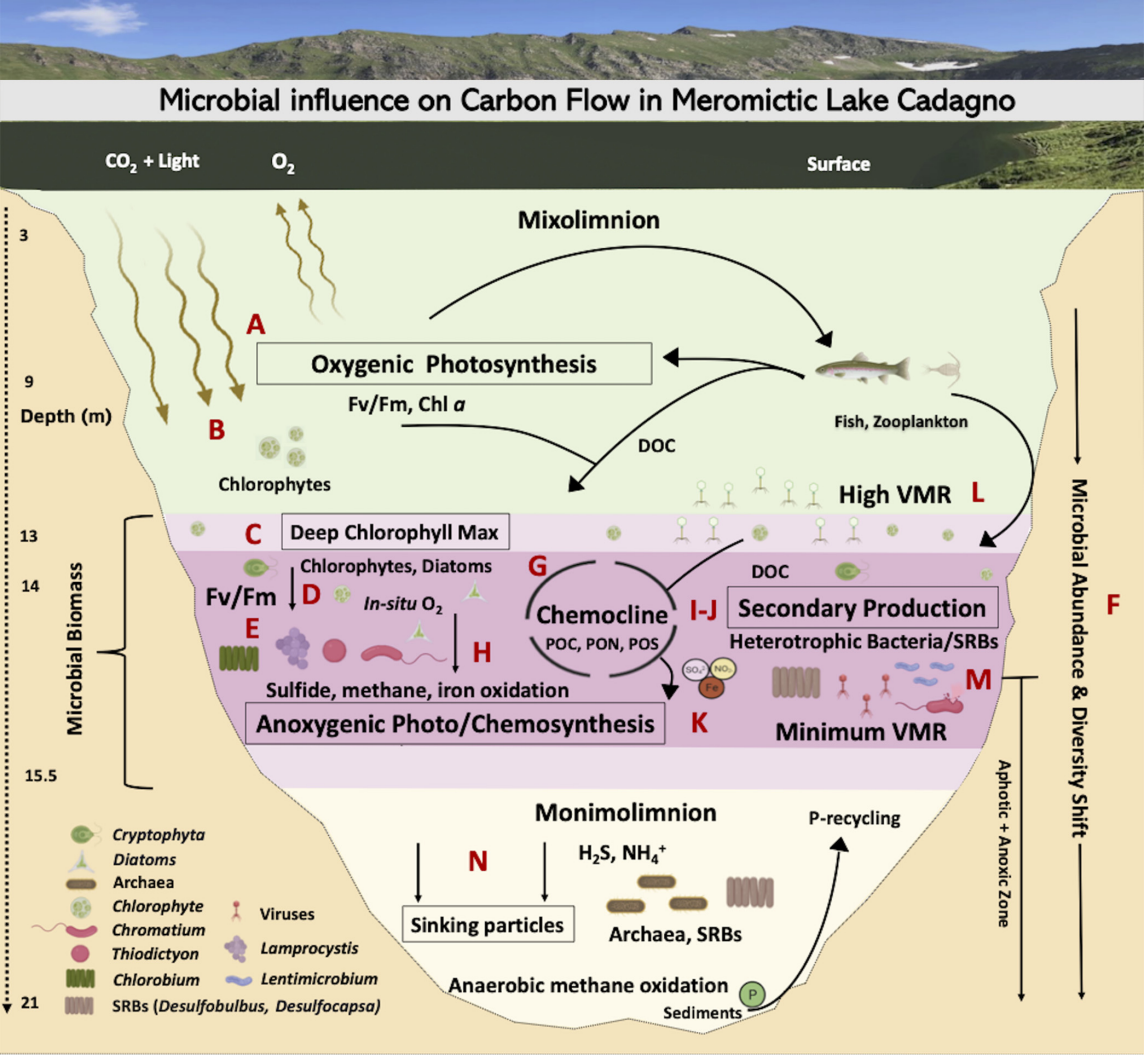
Microbial loop of meromictic Lake Cadagno. Microbiology icons were created with BioRender.

With the major transition to an oxygenated ocean 800 to 700 millions of years ago, the ever-reducing levels of toxic sulfide and relief of nitrogen limitations likely facilitated the evolution of modern eukaryote precursors, as proposed in the Proterozoic ocean model (2, 49). In the oxic mixolimnion of Lake Cadagno, we observed low levels of sulfur and ammonia in combination with high abundances of eukaryotic algal chloroplast OTUs and photosynthetic activity simultaneously with the low abundances of small (<40-μm) prokaryote-like particles. These patterns reflected the conditions predicted during the early period of eukaryotic evolution in the Proterozoic ocean (Fig. 7A). The chloroplast sequences identified in the mixolimnion were dominated by close relatives of the freshwater chlorophyte *Ankyra judayi* (OTU00008) (Fig. 7B), which has not yet been described in Lake Cadagno. This first report of *Ankyra judayi* in Lake Cadagno may reflect the dearth of studies focusing on the eukaryotic phytoplankton composition of the Lake Cadagno mixolimnion, especially those using modern molecular techniques. Likely due to its ability to endure high UV radiation, *Ankyra judayi* has been observed to outcompete other phytoplankton and to grow to high densities in a high-alpine Andean lake (50). These traits may explain the evidence for its presence in high-alpine Lake Cadagno in the sun-lit mixolimnion during the month of August, when UV intensity is known to peak (51), and may reflect traits of early photosynthesizing eukaryotes. Our findings are consistent with prior microscopy-based studies reporting that the majority of the algal biomass was attributed to eukaryotic algae in the Lake Cadagno mixolimnion (20). Moreover, eukaryotes also persisted in the anoxic zone (15 to 19 m), however, *F_v_*/*F_m_* data suggested that they were not photosynthetically active and their presence may be explained by settling from photic zones. These observations of the Lake Cadagno mixolimnion provide one possible model for the period of early evolution of eukaryotes in the oxic strata of the Proterozoic ocean, where, though spatially segregated, they may have cooccurred with abundant and productive chemocline and monimolimnion bacteria.

### Revisiting the microbial linkages between *in situ* oxygen production and anoxygenic processes in the chemocline.

The processes of the major microbial oxygenic and anoxygenic guilds have been reported to contribute almost equally to organic matter production in the Lake Cadagno chemocline (20), as they may have in ancient ocean chemoclines. We observed the coexistence of oxygenic (chlorophytes, diatoms, cyanobacteria) and anoxygenic (PSB and GSB) phototrophs in the Lake Cadagno chemocline (Fig. 7C to E); however, unlike previous conclusions (33, 51), cyanobacterial 16S amplicon reads were rare, while those of chloroplasts of chlorophytes and stramenopiles were abundant in this study. The presence of chlorophytes and stramenopiles in the Lake Cadagno chemocline were consistent with previous studies that indicated *in situ* oxygen production by photosynthetic algae and diatoms (52, 53). Given that eukaryotes also contain phycobilisomes (54) and the abundance of chloroplast 16S rRNA reads of eukaryotic algae observed here, we propose that previous conclusions that attributed phycobilin-based cell counts to cyanobacterial blooms (33, 51) be revisited. Future studies should investigate the relative contribution of cyanobacteria and eukaryotic phytoplankton to oxygenic photosynthesis in the chemocline.

Besides oxygenic and anoxygenic primary production, aerobic respiration has also been proposed to be coupled with biogeochemical processes of the ancient ocean chemocline (2). However, evidence for which microbes are associated with such activity in Proterozoic analogs is sparse. Though typically thought of for its role in photoautotrophy, *Chromatium*, the dominant chemocline microbe in this study, is also known to perform light-independent chemolithoautotrophy and has been reported to contribute up to 40% of total dark carbon fixation through aerobic sulfide oxidation (53) (Fig. 7L). In that study, aerobic respiration by Chromatium okenii cooccurred with *in situ* production of oxygen by photosynthetic algae (53), methane oxidation by methanotrophs. and iron oxidation likely by *Chlorobium* and *Rhodobacter*, where iron oxidation accounted for 10% of total primary production in the chemocline (52, 55) (Fig. 7H). Through their oxygen production, the oxygenic phototrophs (chlorophytes, diatoms, cyanobacteria) observed in the chemocline may play a critical role by enabling these oxidative metabolic processes central to Lake Cadagno’s biogeochemical cycling. These observations indicate that the ancient purple sulfur bacteria may have likely adapted the aerobic metabolism with the rise in oxygenic phototrophs following a great oxygenation event and support the proposal of a mixed community of oxygenic and anoxygenic primary producers in the chemocline of the Proterozoic ocean model (2). Future work targeting the ecological and metabolic interactions between chlorophytes, diatoms, cyanobacteria, and chemocline bacteria at the oxic-anoxic interface of Lake Cadagno will improve our understanding of the evolution of life in ancient oceans.

### Potential for nitrogen metabolism by phototrophic sulfur bacteria and photosynthetic microbial eukaryotes in Proterozoic oceans.

The nitrogen cycle likely mediated by N_2_-fixing cyanobacteria and purple and green sulfur bacteria has been proposed to provide inorganic nitrogen essential for ancient ocean photoautotrophy (2, 12). The shift of oxygenic to anoxygenic photosynthesis observed in this study was coincident with a decreasing efficiency of oxygenic photosynthetic microbes (*F_v_*/*F_m_*), increasing concentrations of hydrogen sulfide, and a shift in bacterial abundance and beta diversity (Fig. 7F). Among the anoxygenic phototrophic sulfur bacteria, Chromatium okenii has been found to perform >80% of the average bulk N_2_ fixation (12) and 40% of the ammonia uptake (19) in the chemocline of Lake Cadagno. The highest abundance of *Chromatium* at the peak of PON and turbidity observed here suggested the potential for ongoing N fixation and nitrogen assimilation in the Lake Cadagno chemocline. In addition to *Chromatium*, other phototrophic purple sulfur bacteria (*Lamprocystis* and *Thiodictyon*) and green sulfur bacteria (*Chlorobium*) are also capable of nitrogen fixation and assimilation (12, 15, 19). These genera are also observed in this study but in low abundances compared to *Chromatium.* While N_2_ fixation is important, ammonia assimilation has been considered to be the major N source, capable of sustaining ~80% of autotrophic carbon fixation in the Lake Cadagno chemocline (12). The eukaryotic phytoplankton observed in this study may also be capable of nitrogen assimilation (56); however, direct evidence for this in Lake Cadagno is lacking. Overall, the diazotrophic purple (*Chromatium*, *Lamprocystis*, and *Thiodictyon*) and green (*Chlorobium*) sulfur bacteria, along with the microbial eukaryotes (chlorophytes), may have assimilated the upwardly diffusing ammonia and facilitated the nitrogen needed for organic matter production (POC, PON, PS) in the Lake Cadagno chemocline (Fig. 7G). Such relief from nitrogen limitation may have supported the persistence of PSB, GSB, and chlorophytes in the permanently stratified Proterozoic ocean (57, 58).

### Nutrient remineralization through secondary production contributes to chemocline biomass.

While it is broadly recognized that the PSB and GSB that dominated the Lake Cadagno chemocline in this study are largely responsible for anoxygenic primary production in this stratum (Fig. 7E) (14, 59), we found that as with primary production, secondary production rates, reported here for the first time, were the highest in the chemocline, exceeding secondary production in the mixolimnion and monimolimnion by 42.4- and 1.9-fold, respectively (Fig. 7I). Here, we estimated that the N generated by secondary production can contribute on average 8.05% ± 1.06% (standard deviation [SD]) of the autotrophic N demand in the chemocline, while N_2_ fixation has been reported to support up to 7.3% (12). In summary, the observations of peak secondary production in the zone of greatest biomass suggested that remineralization is important for supplying nutrients to the active chemocline microbial assemblage.

By pairing these secondary production rates with a description of heterotrophic populations present, we shed light on the clades that may be involved in nutrient remineralization in the chemocline. *Lentimicrobium* was the second most abundant genus in this study and has been previously implicated in fermentation under limited light and oxygen (36). Sulfate-reducing bacteria (SRBs) (Fig. 7J), such as the observed *Desulfocapsa* and *Desulfobulbus*, are known to form aggregates with phototrophic PSB (e.g., *Thiodictyon syntrophicum*, Lamprocystis purpurea), and both cooccurred in the chemocline (37, 60, 61). Through secondary production, heterotrophic microbes are likely involved in the decomposition of particulate organic matter (POC, PON) and particulate sulfur (PS) and the supply of inorganic nutrients necessary to carry out primary production and sustaining populations of oxygenic and anoxygenic photoautotrophs in the chemocline (Fig. 7K). Trends in putative autotrophic and heterotrophic population abundances combined with the secondary productivity rates provide evidence for microbial potential toward recycling of organic matter proposed in the Proterozoic ocean model (2, 8, 10, 12).

### Potential for the role of viruses in modulating microbial activity and nutrient cycling.

Viruses serve as top-down controls on host populations and reprogram host metabolism during infection (23, 24, 62). Evidence for viral activity has been observed in ancient cyanobacterial mat analogues, including their potential for resource scavenging through the degradation of host phycobilisomes, a pigment central to photosynthesis (63). The observed colocalization of high VMR and photosynthetically active phytoplankton in the lower mixolimnion and mixolimnion-chemocline transition may be explained by ongoing lytic viral infections of phytoplankton. Viral lysis products may contribute to the DOC peak and support the high secondary production near the DCM (Fig. 7L). Such a scenario was observed in the phytoplankton *Ostreococcus* virus-host system, where host densities were maintained concurrently with continual lysis due to phase switching between resistant and susceptible hosts (64). If this scenario is occurring, the sustained lysis of phytoplankton has the potential to release DOC in a viral shunt near the mixolimnion-chemocline transition. A DOC peak was indeed observed slightly below this depth in Lake Cadagno. However, given that the VMR is a feature that emerges from a number of underlying virus-microbe interactions, viral life history traits, and environmental cofactors (65), the prediction of sustained phytoplankton viral predation at the mixolimnion-chemocline transition requires empirical confirmation.

Previous studies have identified genomic evidence for viral infection of the presumed major carbon assimilators in the chemocline, *Thiodictyon syntrophicum* and Chromatium okenii (14). Genomes of these organisms sequenced from Lake Cadagno contain CRISPR elements (66, 67), which derive from a type of acquired immunity against invading viral and plasmid DNA. This suggests that viruses may play a role in the modulation of carbon and sulfur cycles, as has been proposed in the hydrothermal vent (68) and wetland (69) microbial communities, where viruses have been found to carry genes central to methanogenesis (*mcrA*) and sulfur reduction (*dsrA*, *dsrD*). Active viral lysis also provides a new possible mechanism to explain the observed low abundances of Chromatium okenii, which have previously been attributed to microbial predation (19). Despite genomic evidence suggesting active infection of these populations (66, 67), VLP counts did not rise with PLP counts in the chemocline. Yet, our observation of a minimum VMR (Fig. 7M) and peak microbial abundances in the Lake Cadagno chemocline supported an emerging trend in stratified meromictic lakes, as the same observation was made in meromictic Ace Lake (Vestfold Hills of East Antarctica) (27). Such coincident low VMR and high microbial abundances have been recognized as a hallmark of the power-law relationship proposed to describe the VMR dynamics in aquatic systems (65). We propose viruses as an important component of the Proterozoic ocean microbial community. Population-specific studies of viral infection are needed in Lake Cadagno to understand the impact of viruses on the evolution and function of microbes that underlie its biogeochemistry and to shed light on the roles viruses may have played in the ancient ocean.

### Conclusion.

This work highlights how biogeochemical exchange within microbial guilds may be driving biomass accumulation to support the food web of the permanently stratified Lake Cadagno. Ultimately, the organic matter generated through eukaryotic and bacterial activity and viral remineralization in the chemocline can be made available to zooplankton and fish. The collective microbial biomass, including viruses, will eventually sink to the sediments contributing to the organic matter burial (Fig. 7N) (17, 70). The observed trends suggest a high degree of interconnectedness and ecological importance of microbial guilds in stratified ancient oceans.

This study inspires future research directions toward a better ecological and biogeochemical understanding of Lake Cadagno and similar meromictic ancient ocean analogues. Our community composition and secondary production data suggested that the ecological role and metabolic potential of heterotrophic bacteria that recycle chemocline biomass in the presence of sulfur and ammonia, such as the second most abundant bacterium, *Lentimicrobium*, and their potential toward recycling chemocline biomass were intriguing unknowns. Furthermore, eukaryotic phytoplankton has been known to contribute to the deep chlorophyll maximum of Lake Cadagno’s chemocline for 2 decades (20), yet insights into the cellular machinery that allows them to photosynthesize under limited light remain unknown. Further, while primary producers (phytoplankton, phototrophic sulfur bacteria) and heterotrophic bacteria are central to the realized function of the lake biogeochemistry and ecology, viruses may shuffle the repertoire of genes controlling both microbial guilds, hence modulating biogeochemical (carbon, nitrogen and sulfur) cycles of the lake. Our empirical observations provide a better understanding of the role that microbes of the chemocline (oxic-anoxic boundary) may have played in the Proterozoic transition from the primarily anoxygenic to oxygenic metabolisms that dominate nutrient biogeochemical cycling in modern oceans (2, 71, 72).

## MATERIALS AND METHODS

### Water sample collection and physicochemical profiling.

This study was conducted in Lake Cadagno (21 m deep), a high alpine meromictic lake situated at 1,921 m above sea level in the Southern Alps of Switzerland (73). Due to the extensive number of samples collected and experiments performed for process measurements, sampling was done over two subsequent days. The oxic mixolimnion (0 to 10 m) was sampled on 28 August 2017 (day 1), and the chemocline (11 to 16 m) and monimolimnion (17 to 19 m) were sampled on 29 August 2017 (day 2). Internal waves are known to affect the position of Lake Cadagno’s stratified water masses, though their frequency is unknown (34). To control for this anticipated variability, a number of parameters are collected on both days to identify the depths of the zones, guiding the sampling of the mixolimnion on day 1 and the chemocline and monimolimnion on day 2. Water sampling and characterization of physical (turbidity, temperature, oxygen, conductivity), biological (Chl *a*, phycocyanin), and chemical (H_2_S, NH_4_^+^) parameters of the water column were performed as previously described (18). Water was sampled using a double-diaphragm Teflon pump (Almatec PSG Germany GmbH) connected to an acid-washed low-density polyethylene (LDPE) line deployed from a platform that was anchored above the deepest part of Lake Cadagno (46.55087°N, 8.71152°E).

Vertical lake profiles were determined using an autonomous conductivity-temperature-depth sensor (CTD; Ocean Seven 316 Plus CTD; Idronaut S.R.L.). The CTD recorded pressure (dbar), temperature (°C), and conductivity (mS/cm); dissolved oxygen (mg/L) was measured with a pressure-compensated polarographic sensor. The error in oxygen profiles for day 2 was manually adjusted by referring to day 1 measurements, which indicated approximately zero oxygen within and below the chemocline. CTD profiles from the whole water column (0 to 20 m, days 1 and 2) were used to identify the distribution of the mixolimnion, chemocline, and monimolimnion and guided water sampling strategy. The CTD was equipped with an LI-192 underwater quantum sensor (Li-Cor Biosciences, NE, USA) that continuously recorded photosynthetically active radiation (PAR-W/m^2^) and a TriLux multiparameter algal sensor (Chelsea Technologies, Ltd., Surrey, UK) that measured *in vivo* Chl *a* (μg/L), an indicator of phytoplankton (including eukaryotic autotrophs and cyanobacteria) biomass (74), and phycocyanin (μg/L), a pigment characteristic of cyanobacteria (75).

### Chemical parameters. (i) Dissolved compounds.

Samples for the analysis of dissolved compounds (DOC) were filtered through an AcroPak filter cartridge (Pall) with a 0.8-μm prefilter and a 0.2-μm final filter. Filters were collected in 30 mL acid-washed and pyrolyzed Pyrex tubes filled to the top and supplemented with 100 μL 2 M HCl. Tubes were stored in the dark at 4°C until processed (November 2017; University of the Geneva) using a Shimadzu TOC-LCPH analysis system. Blanks consisting of Milli-Q water were made, and calibration was done using the standard from 0.2 to 5 ppm. Analyses of dissolved ammonium (NH_4_^+^) and hydrogen sulfide (H_2_S) were done on-site at the Alpine Biology Centre (CBA, Piora, Switzerland) with freshly collected water samples using a UV-visible spectrophotometer (DR 3800; Hach) (76).

### (ii) Particulate compounds.

Particulate organic carbon (POC) and nitrogen (PON) and particulate organic sulfur (POS) were collected by filtering 150 to 500 mL of lake water (volume necessary to begin to clog the filter) on precombusted 47-mm GF/F filters (5 h at 550°C) (catalog no. WH1825-047; Whatman, WI, USA), which were then acidified (500 μL 1 M HCL, added twice at a 1-h interval) and dried in an oven (65°C) overnight in acid-washed Pyrex petri dishes. Samples were sealed using Parafilm and aluminum foil until analysis (November 2017, University of Geneva) using an elemental analyzer (2400 series II CHNS/O elemental analyzer; PerkinElmer). Procedural blanks using Milli-Q water were performed (24 to 25 November 2017) in duplicate to measure the background signal, which was used to correct the concentrations of samples. POC, PON, and POS were expressed in mg/L (ppm). Because purple sulfur bacteria also contain inorganic sulfur globules inside their cell (61, 67), particulate organic sulfur (POS) is referred to as particulate sulfur (PS).

### Biological parameters. (i) Primary production.

Primary productivity was determined using incubation with NaH_14_CO_3_ (catalog no. NEC086H005MC; Perkin Elmer, Waltham, MA, USA) (5 mCi, 1 mL in glass ampoule), which was freshly diluted into 4 mL Milli-Q water at pH 9.6 (adjusted using NaOH; Sigma) and added at a dose of 1 mCi/L of lake water, as described previously (77). Incubation was carried out along an incubation chain deployed at six depths for 23 h on 28 to 29 August 2017. Each depth chain consisted of a metallic ring fixed to a rope at the desired depth (3, 5, 7, 9, 11, and 13 m). The ring was surrounded by six arms on a horizontal plane, each of which ended with a bottle holder. Seventy-five-milliliter acid-washed glass bottles were filled to the top with lake water (ca. 100 mL) at the corresponding depth and spiked with ^14^C prior to being deployed on each level of the incubation chain. At each depth, three transparent bottles were incubated for activity measurements at *in situ* natural light intensities, and three amber bottles were incubated for dark community respiration measurements. At the end of the incubation, each bottle was filtered onto 47-mm GF/F filters (catalog no. WH1825-047; Whatman, WI, USA), which were then stored in a plastic petri dish. Petri dishes were placed in a sealed box containing calcium hydroxide (slaked lime) powder, and after the addition of HCl (1 M, 500 μL) onto the filters, inorganic carbon was degassed overnight. The calcium hydroxide captures the degassed ^14^C, which is further treated as solid radioactive waste. Filters were then collected into 20-mL scintillation vials, supplemented with 10 mL of Ultima Gold AB cocktail, and shaken manually. Primary productivity was expressed in micromoles of net carbon fixation per hour either per liter of lake water or per Chl *a*, considering a dissolved inorganic carbon concentration of 1.8 mM C (16).

### (ii) Maximum quantum yield.

The maximum quantum yield (*F_v_/F_m_*) informs the photosynthetic health and biological activity of algae based on photophysiological characteristics of the chlorophyll photosystem II. Maximum quantum yield was determined using a fast repetition rate fluorometer (FRRF; FastOcean PTX coupled to a FastAct base unit; Chelsea Technologies). Water for maximum quantum yield measurements was preconcentrated 10-fold by gently resuspending the cells as the sample passed through a 0.22-μm 47-mm polycarbonate filter (catalog no. GVWP04700; Millipore, Darmstadt, Germany) using a hand pump (pressure below 15 mbar) and a 47-mm filtration unit (catalog no. 300-4000; Nalgene). This step was done to ensure a high enough method sensitivity and has been shown to alter neither Chl *a* nor cell integrity of samples from Lake Geneva (78). The FRRF was used in single turnover mode to record, in a 45-min dark-adapted natural sample, the basal (*F_b_*) and maximal (*F_m_*) fluorescence following exposure to intense light. The sample was then gently filtered on a 0.22-μm, 47-mm filter (catalog no. GVWP04700; Millipore, Darmstadt, Germany) to record the residual fluorescence (*F_r_*). For each sample, *F_b_* − *F_r_* represented the initial dark-adapted fluorescence, *F_0_*, associated with intact cells. *F_v_/F_m_* was then calculated using the ratio (*F_m_* − *F_0_*)*/F_m_.*

### (iii) FCM.

Total cell counts from fresh unpreserved samples collected on-site, as well as 0.22-μm-filtered cell-free Milli-Q water controls, were generated in triplicate on a BD Accuri C6 cytometer (Becton, Dickinson, San José, CA, USA) using two lasers (blue, 488 nm; red, 640 nm) and four fluorescence detectors (lasers, 488 nm: FL1 = 533/30, FL2 = 585/40, FL3 = 670; laser, 640 nm: FL4 = 675/25). Standard deviations of the triplicates were calculated to assess variations and to evaluate the potential for counting biases obtained from the flow cytometry (FCM) cell counts. FL4 detectors were specifically used to detect emission from phycobilin, a pigment proxy for cyanobacteria. Cells were stained with SYBR green I (catalog no. S7563; Molecular Probes, Eugene, OR) at a ratio of 1:10,000 (vol/vol), following incubation for 13 min at *37°*C in the dark (13). A histogram of event counts versus green fluorescence (FL1 > 1,100) allowed quantification of total cells.

VLPs were counted using flow cytometry (79) after prefiltration of lake water through 55-μm mesh to remove higher organisms such as zooplankton (20), and the lake water was subsequently passed through 0.22-μm filters to remove large particles and cells. To discern viral particles from cell-sized particles or instrument background, 0.22-μm- and 0.02-μm-filtered Milli-Q water samples were used as cell- and virus-free controls, respectively. In triplicate, a 1-mL sample from the filtrate was fixed by adding 10 μL of glutaraldehyde (25% stock solution) in sample cryovials. The samples were fixed for 15 min at room temperature, subsequently stored in liquid nitrogen for shipping, and then stored at –80°C until they were processed in February 2018. Free virus-like particle counts were obtained using a FACSCalibur flow cytometer with a 15-mW 488-nm laser (Becton, Dickinson Biosciences, Grenoble, France). Samples were thawed at 37°C and then diluted in autoclaved and 0.02-μm-filter-sterilized Tris-EDTA (0.1 mM Tris-HCL and 1 mM EDTA, pH 8). Samples were then stained for 10 min at 75°C using SYBR green I (Molecular Probes) at a 1:10,000 final concentration (80). Standard deviations were calculated to assess the potential bias resulting during VLP counting.

### (iv) Phenotypic alpha diversity of PLPs.

FCM-based phenotypic fingerprints have been proven to be a rapid, inexpensive, and robust method to spatially and temporally assess microbial community dynamics (44) and have been considered effective in assessing microbial diversity in aquatic systems (81, 82). Raw flow cytometry files were used to estimate phenotypic traits (morphology and nucleic acid content) and were transformed and normalized using transform and flowBasis functions in the Phenoflow package (44) (https://github.com/rprops/Phenoflow_package) in R (v4.1.0) (83) and RStudio (v1.4.1106) (84). Briefly, the height values of the two fluorescence parameters (FL1, FL3) and two scatter parameters (side scatter [SSC], forward scatter [FSC]) recorded for each cell were treated with a hyperbolic arcsine transformation. These phenotypic intensity values were then scaled to a 0 to 1 range. Each parameter was then normalized based on the average maximum FL1-H intensity value over the data set. The cytometry fingerprint represents community structure in terms of the phenotypic attributes of the cells that comprise the community; essentially, any feature that influences the way the laser interacts with passing particles, such as morphology, size, and nucleic acid content, is captured in the phenotypic fingerprint. Using this approach, prokaryotic phenotypic diversity metrics have been shown to be highly correlated with community taxonomic diversity (44). To calculate phenotypic alpha diversity, Phenoflow calculates Hill number diversity indices (*D_q_*) from order 0 to 2, where 0 represents the richness and *q* > 1 represents richness and evenness, also referred to as D0 (richness), D1 (Shannon), and D2 (inverse Simpson) (85). Phenotypic beta diversity was estimated from the phenotypic feature vectors using the Bray-Curtis distance metric, and the beta diversity ordination was then calculated using nonmetric multidimensional scaling. Fluorescence detectors (lasers, 488 nm: FL1-H = 533/30, FL3-H = 670) were used to target PLP diversity. Polygon gating was applied to filter out the noise observed in control samples (see Fig. S1 in the supplemental material). Clockwise, the polygon gate coordinates (*x*, *y*) were as follows: (7, 6), (15, 6), (15, 17), and (7, 10). Kernel density estimates were calculated by applying a binning grid (128 × 128) to FSC-H (forward scatter height), SSC-H (side scatter height), FL1-H, and FL3-H. The obtained kernel densities values were assigned into one-dimensional vectors, termed “phenotypic fingerprints.”

10.1128/mbio.00052-22.2 FIG S1 Scatter plot of flow cytometry events visualized with a BD6 flow cytometer interface (A and B) and RStudio Phenoflow package (A to D) for one replicate of the 1-m sample. FCM events representing prokaryote-like particles (PLPs) using automatic gating via a four-quadrant layout (A) and manual gating (B and C). (D) FCM events in 0.2-μm-filtered, cell-free Milli-Q water (control). The red outline represents the gating strategy for cell counts and phenotypic diversity analysis. This gate was applied to all samples. Download FIG S1, PDF file, 1.4 MB. Copyright © 2022 Saini et al. 2022 Saini et al. https://creativecommons.org/licenses/by/4.0/ This content is distributed under the terms of the Creative Commons Attribution 4.0 International license.

### (v) Secondary production.

Heterotrophic bacterial production, also termed secondary production, was estimated by the microcentrifuge [^3^H]leucine bacterial production method (86). A working solution of 5 μCi mL^−1^ leucine was made from a manufacturer’s stock solution of radioactive leucine, l-[3,4,5-^3^H(N)]-leucine (catalog no. NET460A005MC; PerkinElmer, MA, USA). To reduce the potential for autodegradation of the leucine radiolabel, stock [^3^H]leucine material was stored in the dark at 2 to 4°C (never frozen) and used for the incorporation experiment within 5 days. Two replicates and one trichloroacetic acid (TCA)-killed control (5% [vol/vol] final concentration) (catalog no. 91228; Sigma) were used for each depth. Briefly, 1.5 mL of lake water samples was incubated with a mixture of radioactive and nonradioactive leucine at final concentrations of 20 nM. Using the [^3^H]leucine working solution, enough “hot” leucine was added to each tube to create a sufficient signal (50% or 85% of hot leucine was used in the mixture for samples collected above and below 13-m depths, respectively). Incubations were conducted for 2 h in a dark incubator at *in situ* temperatures that corresponded to their sample depths. The saturation of leucine incorporation over this period was tested with 20, 30, and 40 nM total leucine. At the end of the incubation, 200 μL of 50% TCA was added to all but the control tubes to terminate leucine incorporation. To facilitate the precipitation of proteins, bovine serum albumin (BSA; Sigma) (100 mgL^−1^ final concentration) was added, and then samples were centrifuged at 16,000 × *g* for 10 min (87). The supernatant was discarded, and the resultant precipitated proteins were washed with 1.5 mL of 5% TCA by vigorous vortexing and again centrifuged (16,000 × *g* for 10 min). The supernatant was discarded. Subsequently, 1.5 mL of UltimaGold LLT (part no. 6013681; PerkinElmer, MA, USA) was added to each vial, mixed, and allowed to sit for >24 h at room temperature before the radioactivity was determined using a liquid scintillation counter (Beckman LS6500). To calculate biomass production, the following conversion factors were applied: molecular weight of leucine of 131.2 g/mol, the fraction of leucine per protein of 0.073, the ratio of cellular carbon to the protein of 0.86, and 2.22 × 10^6^ dpm/μCi to convert disintegrations per minute (the measure of radioactivity) to μCi. A factor of 1.55 kg C mol leucine^−1^ was used to convert the incorporation of leucine to carbon equivalents, assuming no isotope dilution (88).

The contribution of remineralization toward autotrophic N demand was calculated using the following equations:

(1) BR = (BP/BGE) − BP

where *BR* is bacterial respiration proxy for remineralization, *BP* is bacterial production, *BGE* is bacterial growth efficiency (=0.26) (89), and bacterial respiration is expressed in mol C L^−1^ day^−1^.

(2) N generated by heterotrophic bacteria = BR/C:N ratio

where *BR* is bacterial respiration proxy for remineralization, C:N ratio is the ratio of particulate organic carbon (POC) to particulate organic nitrogen (PON), and N generated by heterotrophic bacteria is expressed in μmol N L^−1^ day^−1^.

(3) $$\text{Autotrophic}\;N_{\text{demand}}=\;\left(\text{C fixation rate}/\mathrm{[C}\;:\;\text{N ratio]}\right)$$

where the average C fixation rate between a depth of 13.7 to 15.5 m is 31.1 μmol C L^−1^ day^−1^ (12), the C:N ratio is the ratio of particulate organic carbon (POC) to particulate organic nitrogen (PON), and the autotrophic N demand is expressed in μmol N L^−1^ day^−1^.

(4) Contribution of N remineralization towards autotrophic growth (%) = N generated by heterotrophic bacteria (equation 2)/autotrophic N demand (equation 3)

### Statistical analyses.

The magnitude and significance of relationships between physical and biological parameters were determined with linear regressions and visualized with scatterplots using R (v4.1.0) (83) in RStudio (1.4.1106) (84) using ggplot2 (3.3.3) (90) and ggpubr (0.4.0) (91) packages (92). An *R* of >0.70 was considered a strong positive correlation, and a *P* value of <0.05 was considered statistically significant.

### Illumina MiSeq 16S rRNA gene amplicon sequence analyses. (i) 16S rRNA gene amplicon sequencing.

Water was collected from 3.0, 5.0, 7.0, 9.0, 11.0, 13.0, 15.0, 15.5, 17.0, and 19.0 m (20 L per depth). One sample from each depth was collected for 16S sequencing, representing five from the mixolimnion, three from the chemocline, and two from the monimolimnion. As described in prior studies of Lake Cadagno (20), samples were prefiltered with a 55-μm filter to remove large particles and organisms, such as zooplankton. Using a peristaltic pump, the subsequent biomass was captured on 0.22-μm filters (catalog no. GPWP14250; 142-mm Express Plus filter; Millipore, Darmstadt, Germany) as previously performed for bacterial studies in Lake Cadagno (33, 37, 93, 94). Filters were flash-frozen in liquid nitrogen and stored at –80°C until extraction of DNA by use of a DNeasy blood and tissue kit (catalog no. 69504; Qiagen, Germantown, MD, USA) combined with a QIAshredder (catalog no. 79654; Qiagen, CA, USA), as described previously (95). The first elutions of DNA (50 μL each) were stored (4°C) until sequencing. Dual indexed primers were used, and the V4 region of the 16S rRNA gene amplicon was targeted and amplified, as described previously (96). Sequencing libraries were prepared using the Illumina Nextera Flex kit (Illumina, CA, USA) and sequenced using the MiSeq platform (500 cycles; Illumina) at the University of Michigan Microbiome Core facility.

The sequenced reads were quality controlled (mothur, minlength = 240, maxhomop = 8, maxlength = 275), assembled, trimmed, aligned using SILVA v132 16S rRNA reference sequences (mothur, reference position start = 1968, end = 11550). Following the removal of chimers, sequences were clustered at 97% identity into representative operational taxonomic units (OTU) using mothur (97). Samples that resulted in fewer than 5,000 reads were resequenced to ensure that the full profile was captured with at least two replicates per stratified zone (i.e., mixolimnion, chemocline, and monimolimnion). The OTUs were taxonomically classified using SILVA 132 (98) and TaxAss freshwater (version 1) (99) 16S rRNA gene databases.

### (ii) Microbial community analysis.

Downstream community analysis was carried out by the phyloseq package (100) using R (4.1.0) (83) in RStudio (1.4.1106) (84) to analyze the genotypic alpha and beta diversity of prokaryotic communities. Bacterial and archaeal taxa were merged using merge_phyloseq. To minimize the impact of rare taxa, taxa not observed at least three times in 20% of the samples were removed using the filter_taxa function in phyloseq. OTU counts were transformed to relative abundance (0 to 1 range) by transform_sample_counts. OTUs were not rarefied to preserve rare OTUs for diversity analyses (101). For genotypic beta diversity PCoA (16S amplicon data), OTU counts were Hellinger transformed with the microbiome R package (1.15.3) (102).

To resolve the challenges associated with compositional relative abundance data (103, 104), “inferred absolute abundances” were calculated by multiplying relative abundances by flow cytometry (FCM)-generated cell counts (35) for each sample. Briefly, the relative abundances of prokaryotic OTUs of a given taxon (taxon read count/total read count in sample) were multiplied by the PLP concentrations (cells/mL) for that sample, as determined by flow cytometry. Scaling the 16S amplicon read counts by flow cytometry cell counts provided a more accurate assessment of changes in microbial populations through the water column. This is most strongly demonstrated in the differences observed between Fig. 3A and B. When the total cell counts increase in the chemocline, the magnitude of the changes in populations can be represented only when reads are scaled by cell counts (Fig. 3B).

### (iii) Chloroplast 16S rRNA gene phylogeny.

The Alignment, Classification, and Tree (ACT) functions of SILVA were used to generate the phylogenetic tree of the representative 16S rRNA gene amplicon sequences of putative chloroplast and nonchloroplast OTUs that had been assigned as *Cyanobacteria* (105). Representative sequences of chloroplast OTUs were aligned to the SILVA 132 sequences (SINA 1.2.11). Two neighbors per query sequence (95% minimum sequence identity) were added to the tree with the chloroplast OTU sequences. The advanced variability profile was selected as “auto,” and sequences above 90% identity were kept in order to consider only the highest-confidence, most-homologous sequence. The phylogenetic tree of the OTUs was bootstrapped with RAxML (version 8) (106) with 20 maximum likelihood (ML) and 100 bootstrapped searches. The tree was annotated using the R package ggtree (2.2.4) (107) and Adobe Illustrator (25.2.1).

### Data availability.

Raw sequences were deposited in the NCBI Short Read Archive (SRA) and are available under BioProject accession no. PRJNA717659. The code used for running the mothur analysis, statistical analyses, and figure generation is available in the project GitHub repository at https://github.com/DuhaimeLab/Lake_Cadagno_microbial_loop_Saini_et_al_2021.
