# Supplementary figures and images for: Bacterial, Phytoplankton, and Viral Distributions and Their Biogeochemical Contexts in Meromictic Lake Cadagno Offer Insights into the Proterozoic Ocean Microbial Loop
Source: mBio. 2022 Jun 21;13(4):e00052-22. doi: 10.1128/mbio.00052-22 (PMC9426590; doi:10.1128/mbio.00052-22)

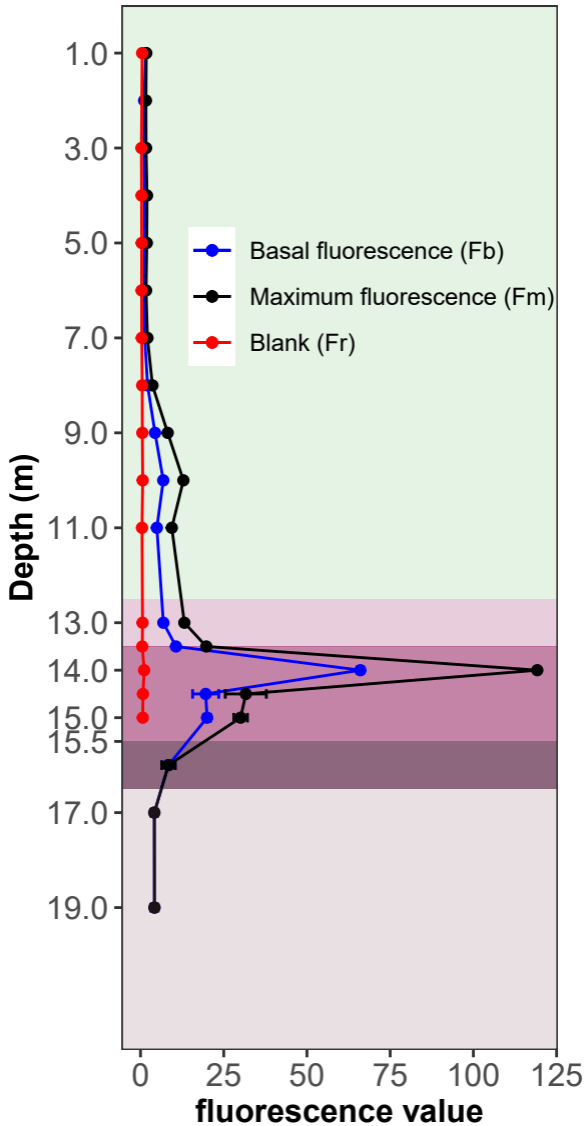

Supplement: FIG S2 [file mbio.00052-22-s0003.pdf]

# Prokaryotic Inferred Absolute Abundance (Genus)

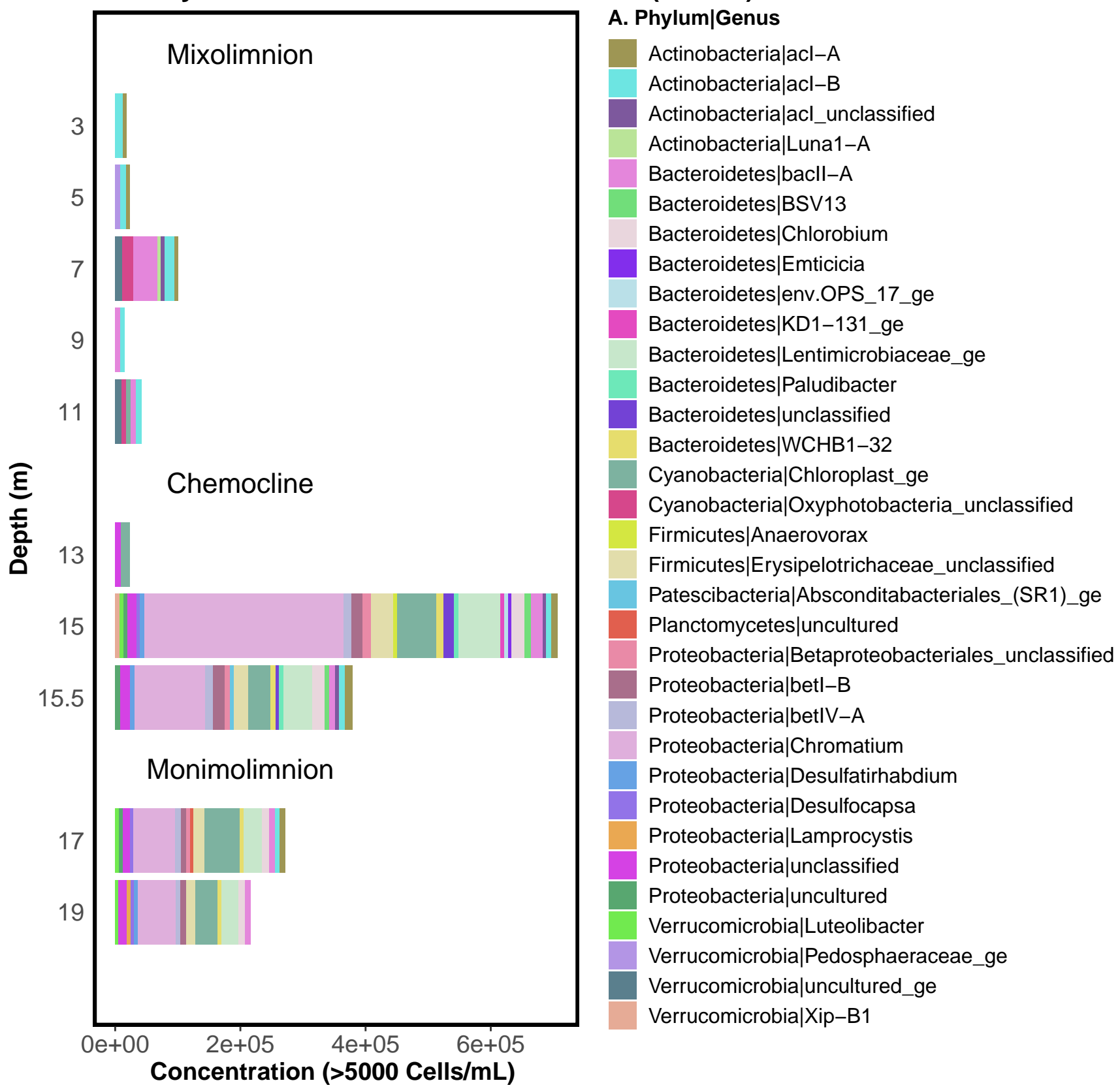

Supplement: FIG S4 [file mbio.00052-22-s0005.pdf]
