# Supplementary material for: Bacterial, Phytoplankton, and Viral Distributions and Their Biogeochemical Contexts in Meromictic Lake Cadagno Offer Insights into the Proterozoic Ocean Microbial Loop
Source: mBio. 2022 Jun 21;13(4):e00052-22. doi: 10.1128/mbio.00052-22 (PMC9426590; doi:10.1128/mbio.00052-22)
Supplement: FIG S1 [file mbio.00052-22-s0002.pdf]

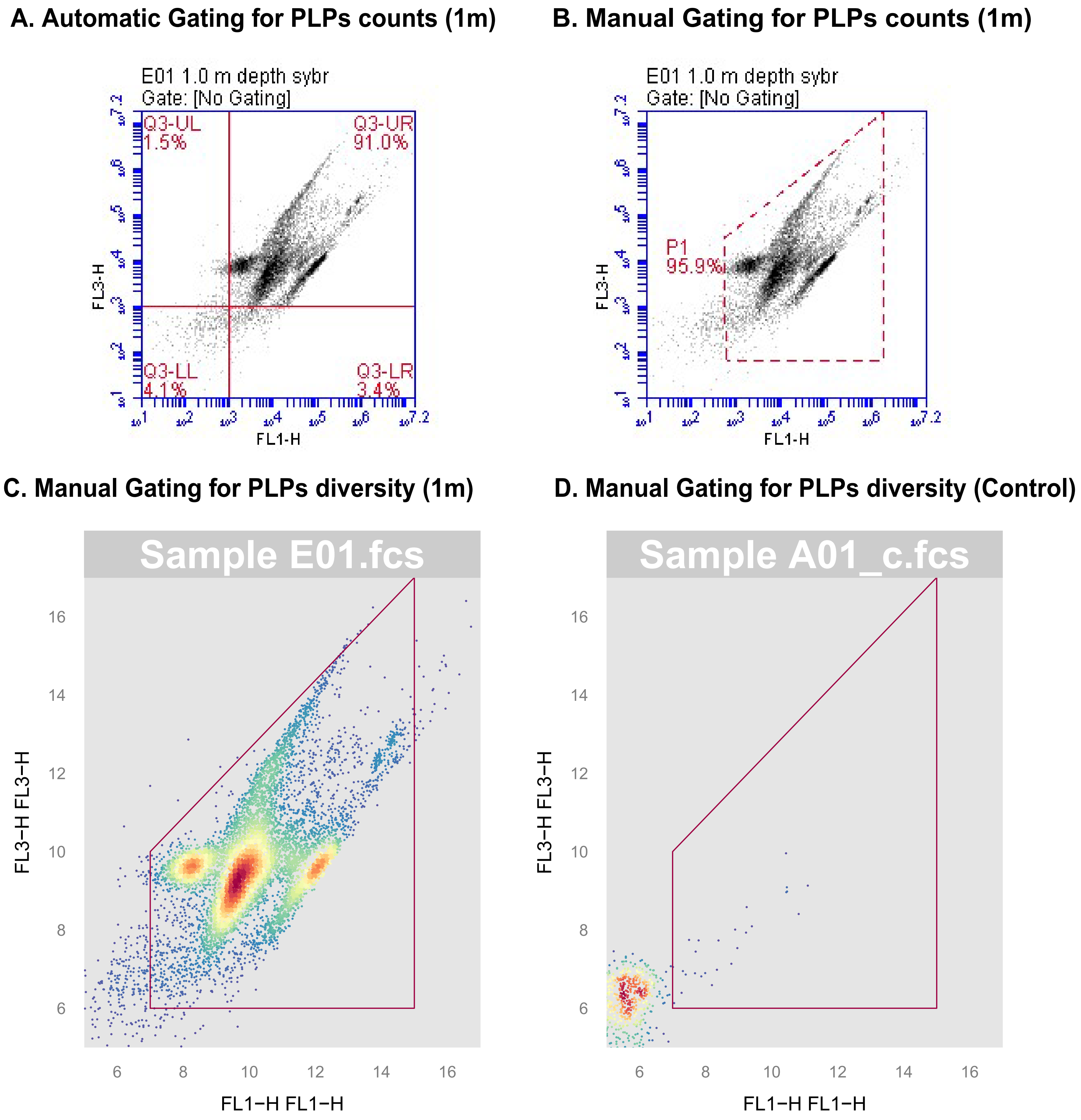
A. Automatic Gating for PLPs counts (1m)
B. Manual Gating for PLPs counts (1m)
E01 1.0 m depth sybr
Gate: [No Gating]
Q3-UL
1.5%
Q3-UR
91.0%
Q3-LL
4.1%
Q3-LR
3.4%
FL3-H
FL1-H
E01 1.0 m depth sybr
Gate: [No Gating]
P1
95.9%
FL3-H
FL1-H
C. Manual Gating for PLPs diversity (1m)
D. Manual Gating for PLPs diversity (Control)
Sample E01.fcs
Sample A01_c.fcs
FL3−H FL3−H
FL1−H FL1−H
FL3−H FL3−H
FL1−H FL1−H
